# Computational Prediction of Compound–Protein Interactions for Orphan Targets Using CGBVS

**DOI:** 10.3390/molecules26175131

**Published:** 2021-08-24

**Authors:** Chisato Kanai, Enzo Kawasaki, Ryuta Murakami, Yusuke Morita, Atsushi Yoshimori

**Affiliations:** 1 Data Science Division, INTAGE Healthcare Inc., 2F NREG Midosuji Bldg., 3-5-7 Kawara-Machi, Chuo-ku, Osaka 541-0048, Japan; murakami-r@intage.com; 2 Business Development Division, Advanced Technology Department, INTAGE Inc., Akihabara Building, 3 Kanda-Neribeicho, Chiyoda-ku, Tokyo 101-8201, Japan; morita-ys@intage.co.jp; 3 Institute for Theoretical Medicine Inc., 26-1 Muraoka-Higashi 2-Chome, Fujisawa 251-0012, Japan; yoshimori@itmol.com

**Keywords:** orphan GPCR, virtual orphan GPCR, enrichment factor (EF), area under receiver operating characteristics (AUROC)

## Abstract

A variety of Artificial Intelligence (AI)-based (Machine Learning) techniques have been developed with regard to in silico prediction of Compound–Protein interactions (CPI)—one of which is a technique we refer to as chemical genomics-based virtual screening (CGBVS). Prediction calculations done via pairwise kernel-based support vector machine (SVM) is the main feature of CGBVS which gives high prediction accuracy, with simple implementation and easy handling. We studied whether the CGBVS technique can identify ligands for targets without ligand information (orphan targets) using data from G protein-coupled receptor (GPCR) families. As the validation method, we tested whether the ligand prediction was correct for a virtual orphan GPCR in which all ligand information for one selected target was omitted from the training data. We have specifically expressed the results of this study as applicability index and developed a method to determine whether CGBVS can be used to predict GPCR ligands. Validation results showed that the prediction accuracy of each GPCR differed greatly, but models using Multiple Sequence Alignment (MSA) as the protein descriptor performed well in terms of overall prediction accuracy. We also discovered that the effect of the type compound descriptors on the prediction accuracy was less significant than that of the type of protein descriptors used. Furthermore, we found that the accuracy of the ligand prediction depends on the amount of ligand information with regard to GPCRs related to the target. Additionally, the prediction accuracy tends to be high if a large amount of ligand information for related proteins is used in the training.

## 1. Introduction

Post-genome research has been providing a large amount of omics data on genes and proteins, including genomes, transcriptomes, and proteomes. On the other hand, the development of technologies such as high-throughput screening has led to the accumulation of compound and bioactivity information on a vast number of compounds and drugs. This information is published in public databases such as ChEMBL [1,2,3] and PubChem [4] and are freely available to use. Such bioactivity information between compounds and proteins is also referred to as drug–target interaction (DTI) and in a broad context, simply Compound–Protein interaction (CPI). The research to utilize such data has been been one of the major hot topics in the field of drug discovery. Many drugs affect the human body in the form of drug effects or side effects through interactions with biomolecules such as target proteins. This is why the identification of CPIs is an important issue in drug discovery research. However, accurate and comprehensive identification of CPIs in experiments is almost impossible due to the enormous costs involved. In recent years, various Artificial Intelligence (AI) technologies have been developed to predict CPIs (or DTIs) on a large scale by effectively utilizing the vast amount of bioactivity data that has been accumulated [5,6,7,8,9,10,11,12,13,14].

In the early stages of drug discovery, ligands that act on target proteins are often insufficiently identified or not even identified at all. In addition, we cannot expect to get a lot of information on the three-dimensional structure of target proteins in these cases. The in silico approach does not work well in situations where known active ligands and protein structural information is extremely limited. Nevertheless, in order to move forward with the drug discovery project, we should also consider trying in silico approaches when we want a ligand even if it is less active. In such cases, AI technology for CPI prediction is promising as an in silico approach.

One of the many AI-based methods for predicting CPIs is called the CGBVS technique. This technique is theoretically simpler than other methods, can be implemented without difficulty, and gives sufficient accuracy of prediction. Hamanaka et al. [14] have implemented CGBVS with a deep neural network (CGBVS-DNN) that enabled training of over a million CPIs. Wassermann et al. [7], using a machine learning approach similar to CGBVS, used a limited number of protease targets as an example to test the prediction accuracy for orphan targets. They have concluded that ligand information of nearest neighbors is essential for a good prediction of ligands of orphan targets.

We studied the following two aspects when using the CGBVS technique. The first aspect is how accurate the ligand prediction is for orphan targets. In this study, we focused on the G protein-coupled receptor (GPCR) family, which is an important and data-rich target in drug discovery. Out of the available 243 possible targets, we randomly selected 52 GPCRs. We created 52 machine learning models of CGBVS, omitting all the ligand information for one particular target GPCR per model. That is, we created a virtual orphan GPCR per model and tested whether the model could predict the ligands for that virtual orphan GPCR. We also investigated how the accuracy of ligand prediction for 52 selected virtual orphan GPCRs is affected by the combination of compound and protein descriptors used in the machine learning process.

The second aspect is to examine the conditions and applicability of high prediction accuracy. Here, we first introduced an applicability index which helped us determine whether it is possible to apply CGBVS to true orphan GPCRs.

## 2. Materials and Methods

### 2.1. CGBVS

For the purpose of investigating the relationship between the applicability of the CGBVS method to ligand prediction of orphan GPCRs and the protein kernel, we used SVM instead of Deep Neural Network. The CGBVS technique we used is mostly implemented according to the method studied by Yabuuchi et al. [8], but the machine learning method for Support Vector Machine (SVM) [15] is slightly different from the original CGBVS technique. The reason is that the kernel function part of SVM is clearly divided into a compound-derived part and a protein-derived part for each calculation. The method of calculating this SVM is the same as the method used in the work of Wassermann et al. [7]. Letting ***c*** be the compound vector and ***p*** be the protein vector, we can then express the Compound–Protein interaction vector (CPI vector) ***x*** as their tensor product x=c⊗p. The SVM kernel for the CPI vector can then be expressed by [16]:

(1) $$K\left(x,x^{\prime }\right)=K_{C}\left(c,c^{\prime }\right)\cdot K_{P}\left(p,p^{\prime }\right),$$

where $K_{C}$ and $K_{P}$ are the compound and protein kernels, respectively. Since the compound and protein kernels can be calculated independently, there is no need to explicitly calculate the matrix representation of the tensor product as a representation of the actual CPI vector.

**Figure 1:**
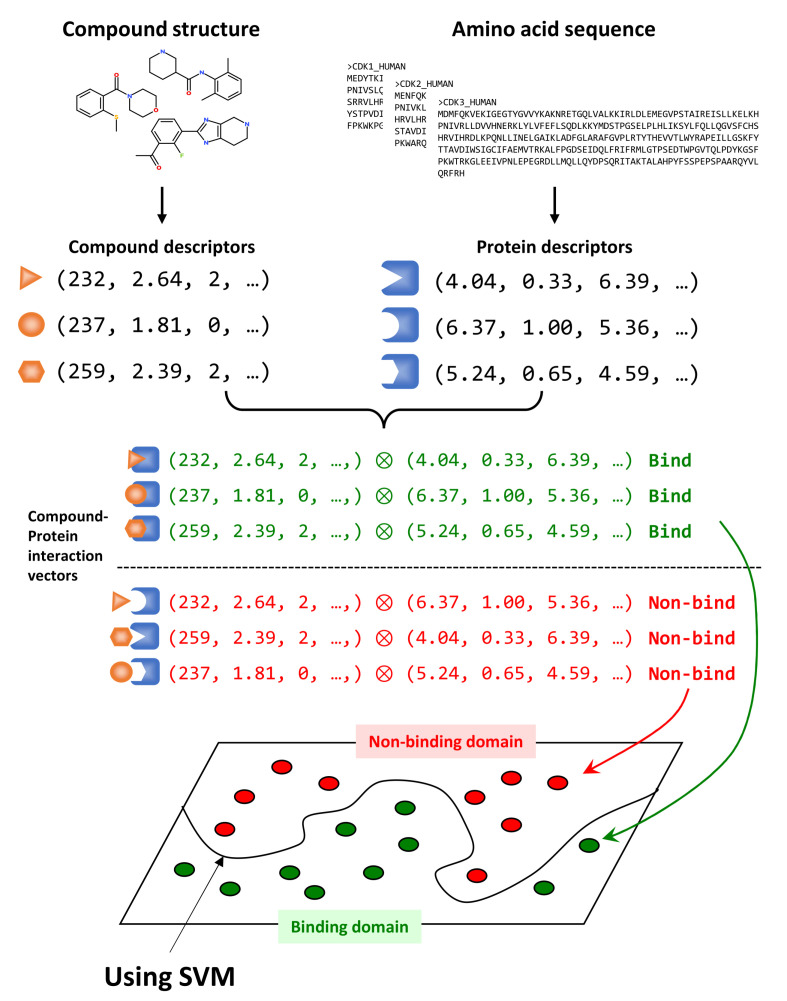
Schematic diagram of how CGBVS is calculated. The feature vector for a compound is obtained by calculating descriptors from the compound’s structural formula. Feature vectors for proteins are calculated from amino acid sequences. The CPI vector is created by taking the tensor product of the compound vector and the protein vector, and is labeled as binding or non-binding vectors based on the activity data in ChEMBL database. The CGBVS model is generated by machine learning of CPI vectors via SVM.

A schematic diagram of the calculation procedure of our CGBVS technique is shown in Figure 1. The first step is to prepare the structural formula data set of the compounds and the amino acid sequence data set of the proteins for machine learning. From a compound structural formula, descriptors such as physicochemical parameters and fingerprints are calculated and converted into a compound vector. From an amino acid sequence, the descriptors associated with the strings are calculated and converted into a protein vector. The two vectors created are combined according to bioactivity data to create the CPI vector. If the activity value of the data is higher than the set threshold, it is a positive CPI vector; otherwise, it is a negative CPI vector. The CGBVS model is created by machine learning via SVM of positive and negative CPI vectors based on aforementioned kernels. This CGBVS model allows us to predict the activity of unknown Compound–Protein combinations. The usual SVM score is the value of the distance from the discriminative surface (decision function), but this value is sigmoidally transformed to perform probability estimation [17].

### 2.2. Virtual Orphan GPCR Model

The CGBVS model developed in this study is based on the GPCR-related activity data from the ChEMBL 25 database [1,2,3]. The total number of GPCRs was 243, and the number of associated compounds was 280,648. The criterion for the presence or absence of activity used in this study was whether there was 50% activity at ≤1μM or at ≥3μM, respectively. In addition, no distinction was made between agonists and antagonists. In addition, data such as the inhibition rate of a single concentration were not used. In this condition, the number of CPIs for positive samples was 165,877 and the number of CPIs for negative samples was 233,272.

**Figure 2:**
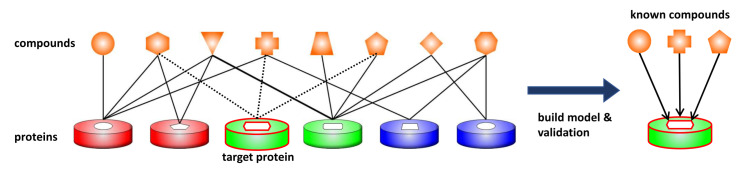
Schematic diagram of the creation of a virtual orphan GPCR model. The solid and dotted lines between the compounds and proteins indicate known activities confirmed from the ChEMBL database. The same solid and dotted lines indicate the Compound–Protein combinations that are used as input to machine learning and the Compound–Protein combinations that are not used as input to machine learning, respectively. Proteins connected by dotted lines indicate a virtual orphan target. The prediction accuracy is verified by screening known GPCR associated compounds.

To create a virtual orphan GPCR model, we select one GPCR and delete the CPI data for that GPCR from the training data set (see Figure 2) Fifty-two GPCRs having 100 or more active ligand data were randomly selected as virtual orphan GPCRs in this study (Table 1).

The CPI data for only one target are deleted per CGBVS model which leaves CPIs for 242 target out of the available 243. As control models to compare the prediction performance of the virtual orphan GPCR models, we also built models that included only half of the original number of ligand data for each target GPCR as a training set and retain the other half as a test set. We refer to these as half-sampled GPCR models (Tables S1 and S2).

The two types of compound descriptors used in this study are descriptors that can be calculated using alvaDesc [18] and ECFP [19]. Using alvaDesc, a software developed by the company Alvascience, 941 non-fingerprint 2D descriptors were calculated, while 2048-bit Extended Connectivity Fingerprints having a radius of 2 (ECFP4) were calculated using RDKit [20]. For proteins, on the other hand, there are three types of descriptors used: PROFEAT 2016 [21], ProtVec [22], and Multiple Sequence Alignment (MSA). PROFEAT descriptors were generated using the web service [23], and we calculated 1437 descriptors using the default settings. ProtVec descriptors were generated using a free tool called BioVec [24] to calculate 1500 descriptors. For MSA descriptors, the number of descriptors generated are equal to the number of target proteins employed in machine learning. There are some techniques that used pairwise sequence alignment as the SVM kernel [25,26]. In our case, we have developed a technique to create descriptors from multiple sequence alignment. To calculate for MSA descriptors, the GPCR amino acid sequences are first prepared in FASTA format. The identity matrix was then generated after performing Multiple Sequence Alignment using Clustal Omega. Then, the identity matrix *S* is eigen-decomposed as in the equation

(2) $$S=U\Lambda U^{T}={\left(\sqrt{\Lambda }U^{T}\right)}^{T}\left(\sqrt{\Lambda }U^{T}\right)=X^{T}X,\;X=\sqrt{\Lambda }U^{T},$$

where Λ is a diagonal matrix and *U* is a unitary matrix made from eigenvectors. Finally, each column of the matrix *X* in Equation (2) can be taken as the column feature vector of the corresponding protein. In rare cases, several eigenvalues with small negative numbers may be found. In such cases, the eigenvalues and eigenvectors of the negative numbers are removed, and the matrix *X* is calculated. The MSA feature vectors computed above can be reconstructed through approximation of the matrix elements of the identity matrix *S* by choosing a linear kernel as the SVM kernel. Since the negative eigenvalues and eigenvectors have been removed, this kernel matrix is a semi-positive definite symmetric matrix.

The compound and protein descriptors described above are high dimensional vectors, thus we used principal component analysis to perform dimensionality reduction. We took the cumulative contribution of the principal components up to 99%.

The kernel function computed in SVM machine learning depends on the feature vector created from each descriptor. Table 2 shows the correspondence between descriptors and kernel functions. In this study, we created virtual orphan GPCR models for all combinations of two compound and three protein descriptors (six combinations) for each of the 52 GPCRs. To avoid overfitting of the SVM model, we have set the appropriate hyperparameters to maximize accuracy via 5-fold cross validation. Machine learning calculations with SVM have been performed using a proprietary tool named CzeekS [27].

### 2.3. Model Validation

As a method of confirming the prediction performance of virtual orphan GPCR models, a set of compounds for validation was screened against the virtual orphan GPCR. The set of compounds used for validation was composed of 280,648 GPCR-related compounds from the ChEMBL 25 database. These compounds are identical to those used to create the CGBVS models, but since each model is created after deleting the data of the GPCR to be tested, they are not considered to be problematic as validation compounds for prediction performance. Validation of HS GPCR models was performed in the same way as the virtual orphan GPCR models; however, the ligand data of the target GPCR included in the training set were omitted from the test set. The area under receiver operating characteristic curve (AUROC) and Enrichment Factor (EF) were adopted as measures of predictive performance. In these calculations, compounds whose interaction data with the target do not exist in the CHEMBL database were treated as having no activity. AUROCs are calculated using scikit-learn by sorting in order of increasing CGBVS score. On the other hand, ${\mathrm{EF}}_{1\%}$ is calculated as

(3) $${\mathrm{EF}}_{1\%}=\frac{\left(A_{\mathrm{found}}/N_{\mathrm{subset}}\right)}{\left(A_{\mathrm{total}}/N_{\mathrm{total}}\right)}.$$

$N_{\mathrm{total}}$ is the total number of compounds screened and $N_{\mathrm{subset}}$ is the number of compounds selected from the top scores. In addition, $A_{\mathrm{total}}$ is the total number of active compounds for the target GPCR, and $A_{\mathrm{found}}$ is the number of active compounds for the target GPCR found among the top scoring compounds selected.

### 2.4. Applicability Index

We considered the applicability index $A(p_{i})$ of CGBVS to the target GPCR $p_{i}$ to be proportional to the sum of the number of active ligands $N_{j}$ of the neighboring GPCRs $p_{j}$ of the target virtual orphan GPCR. Thus, we defined it as

(4) $$A\left(p_{i}\right)=\sum_{j}w\left(K_{P}\left(p_{i},p_{j}\right)\right)N_{j}.$$

Here, *w* is a weight function whose argument is the value of the protein kernel function $K_{P}$, and its functional form is the sigmoid function

(5) $$w\left(x\right)=\frac{1}{1+\exp\left(-\alpha \left(x-r\right)\right)}.$$

The two parameters of the sigmoid function, α and *r*, are determined to maximize the Spearman’s correlation coefficient between AUROC and logA. The AUROC is calculated using the procedure described here earlier. Bayesian optimization was used to perform the optimization of the correlation coefficient.

## 3. Results and Discussion

### 3.1. Analysis of Prediction Accuracy

**Figure 3:**
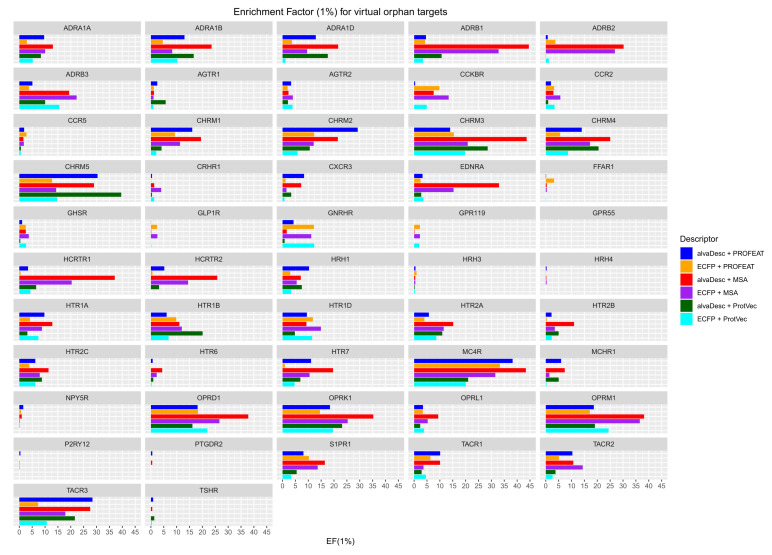
${\mathrm{EF}}_{1\%}$ of the screening calculation results using the virtual orphan GPCR models. Red, blue, and green bars indicate the combination of alvaDesc with MSA, PROFEAT, and ProtVec, respectively. Purple, orange, and light blue bars indicate the combination of ECFP with MSA, PROFEAT, and ProtVec, respectively.

The ${\mathrm{EF}}_{1\%}$ calculated in this study are for the top 1% (2806 compounds) from the highest scores of the screened compounds. In this case, ${\mathrm{EF}}_{1\%}$ takes a value from 0 to 100 and, when ${\mathrm{EF}}_{1\%}$ is equal to 1, it corresponds to random screening. The model is not worthy for screening unless the ${\mathrm{EF}}_{1\%}$ value is at least above 1. The enrichment factors for the 52 GPCRs used as virtual orphan targets are shown in Figure 3. Additionally, a table comparing the values of ${\mathrm{EF}}_{1\%}$ for the virtual orphan and half-sampled GPCR models is provided as Supplementary Data (Table S1). All possible combinations of compound and protein descriptors were tested for each GPCR target and results showed large variations in ${\mathrm{EF}}_{1\%}$ among GPCRs. The same was observed in ${\mathrm{EF}}_{1\%}$ among descriptor combinations for the same GPCR. This may indicate that each GPCR target possibly requires different combination of descriptors that are suitable for accurate prediction. Figure 3 shows that the combination of alvaDesc and MSA (red bars) has good ${\mathrm{EF}}_{1\%}$ values for most GPCRs, indicating that it could possibly be the best descriptor combination. The next best descriptor combination is exhibited by alvaDesc-PROFEAT (blue bars) followed by ECFP-MSA (purple bars).

In order to simplify the ${\mathrm{EF}}_{1\%}$ results for each GPCR and highlight the effect of descriptor combinations, the frequency distribution of ${\mathrm{EF}}_{1\%}$ for each descriptor combination is summarized in Table 3. For all combinations of descriptors, more than half of the GPCRs had ${\mathrm{EF}}_{1\%}$ greater than 1 (more than 26), and some of them had ${\mathrm{EF}}_{1\%}$ greater than 30, which can be considered accurate and better than our expectations. Some of them were comparable to the ${\mathrm{EF}}_{1\%}$ of the half-sampled GPCR models, and, surprisingly, there were six ${\mathrm{EF}}_{1\%}$ values that exceeded those of the half-sampled models. Looking at the variations in ${\mathrm{EF}}_{1\%}$ value and the number of GPCRs for each descriptor, it can be seen that the difference in the protein descriptor has a greater impact on the ${\mathrm{EF}}_{1\%}$ than the difference in the compound descriptor. We have found that, when MSA is used as the protein descriptor, there are many GPCRs having better ${\mathrm{EF}}_{1\%}$ than when other descriptors are used. In particular, for the combination of alvaDesc and MSA, there were nine GPCRs with ${\mathrm{EF}}_{1\%}$ greater than 30, making it the most suitable combination for the construction of the GPCR model.

**Figure 4:**
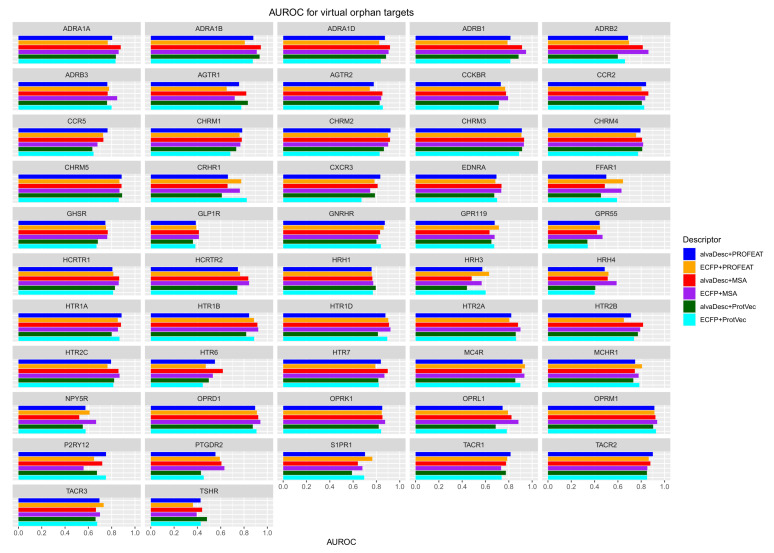
AUROC of the screening calculation results using virtual orphan GPCR models. Red, blue, and green bars indicate the combination of alvaDesc with MSA, PROFEAT, and ProtVec, respectively. Purple, orange, and light blue bars indicate the combination of ECFP with MSA, PROFEAT, and ProtVec, respectively.

**Figure 5:**
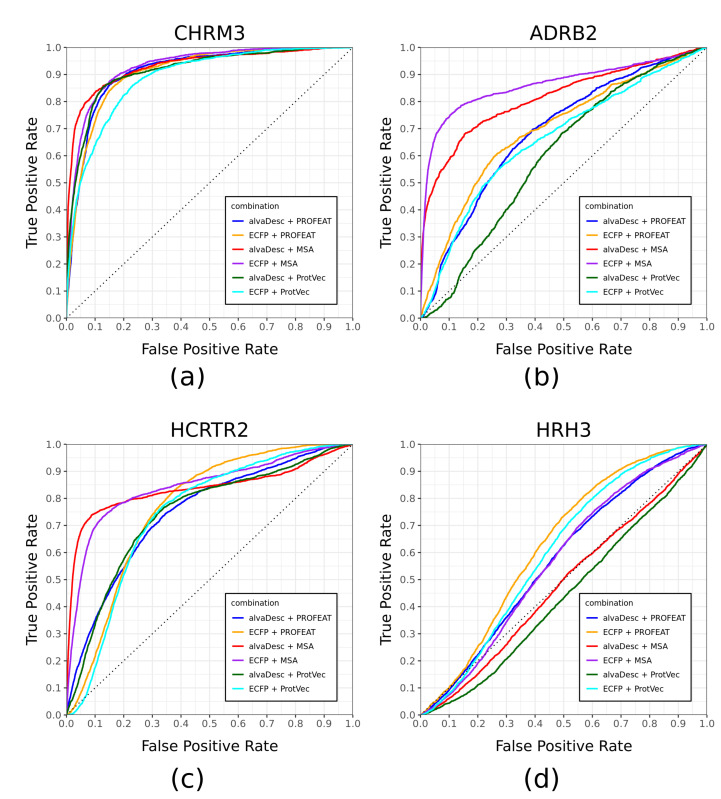
ROC curves of four representative GPCRs generated after screening of designated compound dataset using virtual orphan GPCR models. Characteristic results are shown for four selected GPCRs. Red, blue, and green lines indicate the combination of alvaDesc with MSA, PROFEAT and ProtVec, respectively. Purple, orange, and light blue lines indicate the combination of ECFP with MSA, PROFEAT, and ProtVec, respectively. (**a**) CHRM3; (**b**) ADRB2; (**c**) HRH3; (**d**) HCRTR2.

${\mathrm{EF}}_{1\%}$ is commonly used as a performance indicator for screening measurements. Since the number of active compounds for each protein is different, ($A_{\mathrm{total}}$ in the Equation (3)), care must be taken in the simple comparison of prediction performance between different GPCRs. Therefore, in this study, we calculated AUROC (area under the ROC curve) as another predictive performance indicator. The calculation results of AUROC for 52 GPCRs are shown in Figure 4. As in the case of ${\mathrm{EF}}_{1\%}$, a table comparing the AUROC with the half sampled GPCR models is shown in Table S2. For all combinations of GPCRs and descriptors, the values of AUROC for the half-sampled GPCR models are higher than that for the virtual orphan GPCR model. Figure 5 shows four representative ROC curves from 52 GPCRs. Each one has been chosen for its particular characteristic. CHRM3 is a case where all combinations of descriptors have high predictive performance, while ADRB2 and HCRTR2 are cases where the six curves are scattered. HRH3 is a case where the predictive performance for all combinations of descriptors is low.

For the three GPCRs in Figure 5 that result in high prediction performance, it can be seen that the prediction performance is good in two descriptor combinations: alvaDesc-MSA (red) and ECFP-MSA (purple). The trend is roughly the same for other GPCRs. The number of GPCRs with AUROC greater than 0.8 was the largest in alvaDesc-MSA with 29, followed by ECFP-MSA with 27. The number for other descriptor combinations was 18–24. These results suggest that the best prediction results are obtained when MSA is used as the protein descriptor. It is interesting to note that the number of GPCRs with AUROC of 0.8 or higher in the half sampled GPCR models is 49 for all six combinations of descriptors (see Table S2). This means that the difference in prediction accuracy due to the difference in descriptors is small in the conventional method of accuracy comparison such as cross-validation but is clearly bigger when using virtual orphan GPCR models. Furthermore, in the case of virtual orphan GPCR models, prediction is greatly influenced by the combination of compound and protein descriptor used.

### 3.2. Applicability of CGBVS for Orphan Targets

In actual drug discovery research, when trying to search for active compounds of true orphan GPCRs using CGBVS, it is necessary to have an indicator of whether CGBVS will work or not. According to the results of the performance evaluation of CGBVS models by ${\mathrm{EF}}_{1\%}$ and AUROC, the prediction performance is high for widely studied GPCRs, such as adrenergic and muscarinic receptors, for which there is abundant data on known ligands. This is thought to be because the prediction performance does not deteriorate even if all the ligand data of the target orphan GPCR is deleted, since abundant ligand data of related GPCRs of the target GPCR can be included in the training data. This can be understood from the fact that the applicability domain of a machine learning model is often set to the region around a dense area of training data [28,29,30]. Therefore, we defined the applicability index $A(p_{i})$ of CGBVS to be proportional to the sum of the number of active ligands $N_{j}$ of the related GPCRs $p_{j}$ to the target GPCR, as in Equation (4).

**Figure 6:**
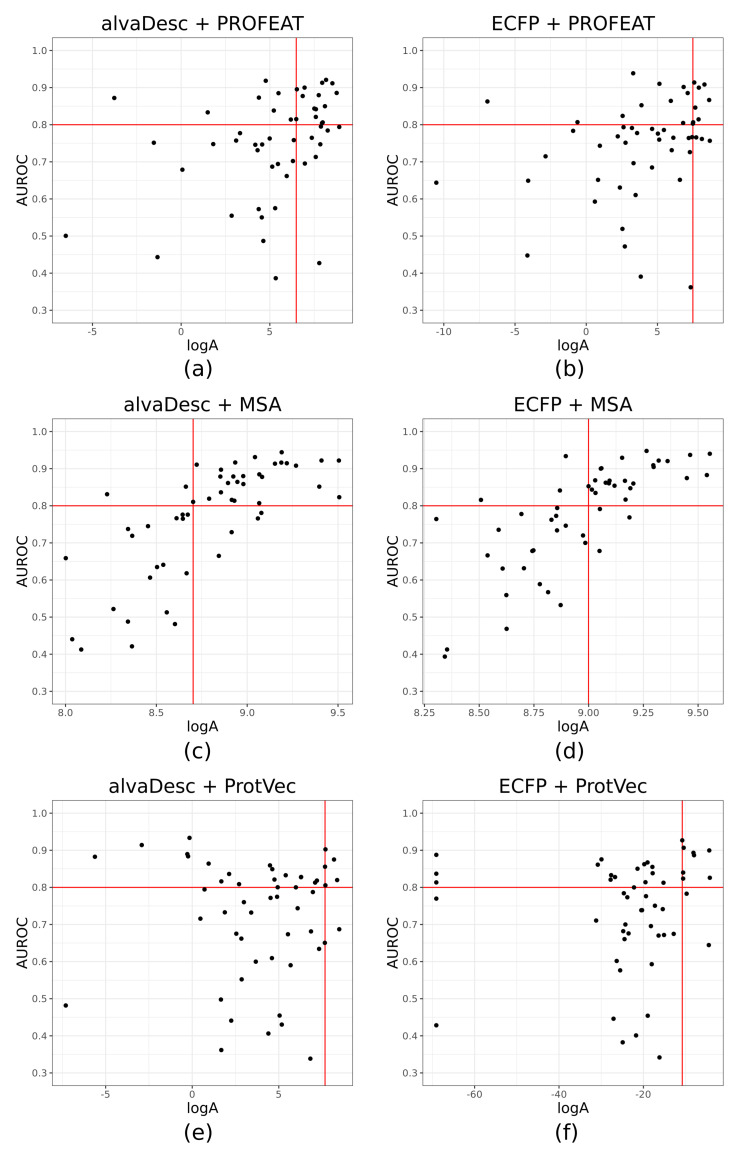
Relationship between applicability index and AUROC for six combinations of compound and protein descriptors. A horizontal red line indicates the AUROC value at 0.8. A vertical red line indicates the threshold value for the applicability index. (**a**,**b**) PROFEAT; (**c**,**d**) MSA; (**e**,**f**) ProtVec.

The results of calculating the applicability index as described above for six different combinations of compound and protein descriptors are shown in Figure 6 as scatter plots. Values of Spearman’s correlation between logA and AUROC are summarized in Table 4. Similar to ${\mathrm{EF}}_{1\%}$, the variation in the values of correlation coefficients are also larger among protein descriptors compared to that among compound descriptors. In addition, the protein descriptor with the highest correlation coefficient is MSA, and both alvaDesc and ECFP are highly correlated with logA and AUROC. As for the other two protein descriptors, PROFEAT showed a weak correlation and ProtVec showed no correlation at all. Therefore, when attempting to find the ligand for a true orphan GPCR using CGBVS, the use of MSA as the protein descriptor can provide some estimate of whether the ligand search will be successful or not. Virtual orphan GPCRs with AUROC greater than 0.8 are positive, and those with AUROC less than 0.8 are negative, and are predicted based on whether they exceed the threshold of logA. The threshold of logA was determined to maximize the accuracy of the prediction. Table 5 summarizes the results of validating the prediction accuracy of virtual orphan GPCRs with AUROC greater than 0.8. The accuracy and positive predictive value (PPV) of MSA was close to 0.9, indicating high prediction accuracy compared to other protein descriptors. In the case of PROFEAT, the accuracy was not bad at over 0.7, but the PPV was a little low at 0.65 when the compound descriptor was alvaDesc.

One of the features of MSA protein descriptor is that the values of the parameters *r* and α of the applicability index weight function are smaller than those of the other two types of protein descriptors. This means that the weight function is looser in shape than the other protein descriptors, and the active ligands of GPCRs with small protein kernel values (small similarity) also contribute to the applicability index. This can be understood from the fact that the threshold of logA is the largest for MSA. In using MSA, although machine learned target proteins are less similar to the virtual orphan target, we were able to construct a machine learning model in which the ligand information exert influences on each other. This leads to high applicability of CGBVS to identifying ligands for orphan targets. The opposite can be said for PROFEAT which requires high similarity between machine learned and orphan targets in order to have high applicability. On the other hand, the weight function in the case of PROFEAT is a parameter with a shape that changes more rapidly at the position where the protein kernel value is larger than MSA. This means that only the ligand information of GPCRs that are very close to the virtual orphan GPCRs affects the prediction accuracy. In a study by Wassermann et al., the accuracy of ligand prediction for orphan targets was found to be greatly influenced by the ligand information of related targets. Our results are consistent with theirs, and Equation (4) gives a more generalized interpretation.

## 4. Conclusions

We tested the prediction accuracy of the CGBVS technique for 52 virtual orphan GPCRs. Machine learning with the CGBVS method was performed for all possible combinations of two types of compound descriptors and three types of protein descriptors. In the prediction of the ligands of virtual orphan GPCRs, it was shown that the protein descriptor had a greater impact on the prediction accuracy than the compound descriptor. Of the three types of protein descriptors validated in this study, MSA had the best accuracy, with the highest number of GPCRs exceeding the reference values (${\mathrm{EF}}_{1\%}>10$, AUCROC>0.8) for both ${\mathrm{EF}}_{1\%}$ and AUROC indices. On the other hand, for compound descriptors, alvaDesc had slightly more GPCRs with better prediction accuracy than ECFP, but with only small differences between actual values.

We also examined the conditions under which ligand search for virtual orphan GPCRs was possible using CGBVS. The simple applicability index we defined in Equation (4) was shown to correlate well with AUROC when an MSA descriptor was used. There is a weak correlation for PROFEAT and almost no correlation at all for ProtVec. By using an MSA descriptor, we can, therefore, determine whether CGBVS can be applied to an unknown orphan target by the value of logA. In this case, if logA is 8.7 or higher when using alvaDesc as compound descriptor, and if logA is 9.0 or higher when using ECFP, a high success rate can be expected.

## Figures and Tables

**Table 1:** List of 52 GPCRs that were selected as virtual orphan targets in this study. In the table, the active column shows the number of ligands that are active (≤1μM), and the inactive column shows the number of ligands that are inactive (≥3μM).

Gene Name	Accession	Active	Inactive	Protein Name
ADRA1A	P35348	1800	244	Alpha-1A adrenergic receptor
ADRA1B	P35368	1425	302	Alpha-1B adrenergic receptor
ADRA1D	P25100	1369	248	Alpha-1D adrenergic receptor
ADRB1	P08588	1021	539	Beta-1 adrenergic receptor
ADRB2	P07550	1542	1832	Beta-2 adrenergic receptor
ADRB3	P13945	1472	215	Beta-3 adrenergic receptor
AGTR1	P30556	1167	599	Type-1 angiotensin II receptor
AGTR2	P50052	900	113	Type-2 angiotensin II receptor
CCKBR	P32239	1014	516	Gastrin/cholecystokinin type B receptor
CCR2	P41597	1379	287	C-C chemokine receptor type 2
CCR5	P51681	1749	333	C-C chemokine receptor type 5
CHRM1	P11229	1768	1088	Muscarinic acetylcholine receptor M1
CHRM2	P08172	1493	663	Muscarinic acetylcholine receptor M2
CHRM3	P20309	1666	605	Muscarinic acetylcholine receptor M3
CHRM4	P08173	751	522	Muscarinic acetylcholine receptor M4
CHRM5	P08912	510	651	Muscarinic acetylcholine receptor M5
CRHR1	P34998	1648	239	Corticotropin-releasing factor receptor 1
CXCR3	P49682	1099	184	C-X-C chemokine receptor type 3
EDNRA	P25101	1195	257	Endothelin-1 receptor
FFAR1	O14842	774	300	Free fatty acid receptor 1
GHSR	Q92847	1541	191	Growth hormone secretagogue receptor type 1
GLP1R	P43220	3452	94,774	Glucagon-like peptide 1 receptor
GNRHR	P30968	1217	96	Gonadotropin-releasing hormone receptor
GPR119	Q8TDV5	1234	110	Glucose-dependent insulinotropic receptor
GPR55	Q9Y2T6	153	553	G-protein coupled receptor 55
HCRTR1	O43613	2200	783	Orexin receptor type 1
HCRTR2	O43614	2611	725	Orexin receptor type 2
HRH1	P35367	999	406	Histamine H1 receptor
HRH3	Q9Y5N1	3395	212	Histamine H3 receptor
HRH4	Q9H3N8	903	318	Histamine H4 receptor
HTR1A	P08908	3532	480	5-hydroxytryptamine receptor 1A
HTR1B	P28222	932	190	5-hydroxytryptamine receptor 1B
HTR1D	P28221	1078	133	5-hydroxytryptamine receptor 1D
HTR2A	P28223	3540	676	5-hydroxytryptamine receptor 2A
HTR2B	P41595	1337	381	5-hydroxytryptamine receptor 2B
HTR2C	P28335	2588	756	5-hydroxytryptamine receptor 2C
HTR6	P50406	2925	306	5-hydroxytryptamine receptor 6
HTR7	P34969	1532	248	5-hydroxytryptamine receptor 7
MC4R	P32245	2311	857	Melanocortin receptor 4
MCHR1	Q99705	3116	524	Melanin-concentrating hormone receptor 1
NPY5R	Q15761	1038	100	Neuropeptide Y receptor type 5
OPRD1	P41143	3180	2086	Delta-type opioid receptor
OPRK1	P41145	3743	1197	Kappa-type opioid receptor
OPRL1	P41146	1305	128	Nociceptin receptor
OPRM1	P35372	3797	2033	Mu-type opioid receptor
P2RY12	Q9H244	912	237	P2Y purinoceptor 12
PTGDR2	Q9Y5Y4	2541	143	Prostaglandin D2 receptor 2
S1PR1	P21453	2165	379	Sphingosine 1-phosphate receptor 1
TACR1	P25103	2334	223	Substance-P receptor
TACR2	P21452	794	227	Substance-K receptor
TACR3	P29371	788	143	Neuromedin-K receptor
TSHR	P16473	1140	15,271	Thyrotropin receptor

**Table 2:** Table of correspondence between compound or protein descriptors and SVM kernel functions.

Descriptor	Class	SVM Kernel	Equation
alvaDesc	compound	Gaussian	$K\left(x_{i},x_{j}\right)=\exp\left(-\gamma {\left(x_{i}-x_{j}\right)}^{2}\right)$
ECFP	compound	Tanimoto	$K\left(x_{i},x_{j}\right)=\frac{x_{i}\cdot x_{j}}{∥x_{i}{∥}^{2}+{∥x_{j}∥}^{2}-x_{i}\cdot x_{j}}$
PROFEAT2016	protein	Gaussian	$K\left(x_{i},x_{j}\right)=\exp\left(-\gamma {\left(x_{i}-x_{j}\right)}^{2}\right)$
ProtVec	protein	Gaussian	$K\left(x_{i},x_{j}\right)=\exp\left(-\gamma {\left(x_{i}-x_{j}\right)}^{2}\right)$
MSA	protein	linear	$K\left(x_{i},x_{j}\right)=x_{i}\cdot x_{j}$

**Table 3:** Enrichment factor values and the number of GPCRs for each combination of descriptors. The compound and protein columns indicate the compound descriptor and protein descriptors used, respectively.

Descriptors		${\mathrm{EF}}_{1\%}$			
**Compound**	**Protein**	**0–1**	**1–10**	**10–30**	**30–50**
alvaDesc	PROFEAT	13	23	14	2
ECFP	PROFEAT	14	28	9	1
alvaDesc	MSA	10	14	19	9
ECFP	MSA	8	19	22	3
alvaDesc	ProtVec	17	20	14	1
ECFP	ProtVec	15	26	11	0

**Table 4:** Spearman’s correlation between AUROC and logA and the parameters α and *r* of the weight function for six combinations of compound and protein descriptors.

Descriptors		Spearman’s Corr.	α	r
Compound	Protein			
alvaDesc	PROFEAT	0.4466	79.73	0.6264
ECFP	PROFEAT	0.3949	94.18	0.6385
alvaDesc	MSA	0.7792	12.77	0.4365
ECFP	MSA	0.8047	11.04	0.4564
alvaDesc	ProtVec	−0.0362	39.87	0.5801
ECFP	ProtVec	0.1759	89.28	0.8000

**Table 5:** Accuracy of predicting GPCRs with an AUROC of 0.8 or higher using the applicability index for six different combinations of compound and protein descriptors. The logA column is the threshold of the applicability index, PPV is the positive predictive value, Accuracy is the prediction accuracy, and *p*-value is the result of Fisher’s exact test.

Descriptors		logA	PPV	Accuracy	*p*-Value
Compound	Protein				
alvaDesc	PROFEAT	6.490	0.6521	0.7115	$4.580\times 10^{-3}$
ECFP	PROFEAT	7.486	0.7272	0.7500	$4.812\times 10^{-3}$
alvaDesc	MSA	8.702	0.8710	0.8846	$1.899\times 10^{-8}$
ECFP	MSA	8.999	0.8889	0.8846	$2.549\times 10^{-8}$
alvaDesc	ProtVec	7.671	0.8333	0.6153	$8.357\times 10^{-2}$
ECFP	ProtVec	−10.79	0.8000	0.6731	$1.542\times 10^{-2}$

## Data Availability

The data presented in this study are available in this article and related supplementary material (Tables S1 and S2).

## Supplementary Materials

The following are available online. Table S1: Enrichment factors for 52 GPCRs which were calculated from the results of screening using virtual orphan (VO) and half-sampled (HS) GPCR models, Table S2: AUROC for 52 GPCRs which were calculated from the results of screening using virtual orphan (VO) and half-sampled (HS) GPCR models.
