# Supplementary material for: Computational Prediction of Compound–Protein Interactions for Orphan Targets Using CGBVS
Source: Molecules. 2021 Aug 24;26(17):5131. doi: 10.3390/molecules26175131 (PMC8434178; doi:10.3390/molecules26175131)
Supplement: Supplementary file 1 [file molecules-26-05131-s001.zip › supplement.pdf]

Supplementary Materials: Computational prediction of compound-protein interactions for orphan targets using CGBVS

Chisato Kanai [1]*, Enzo Kawasaki [1], Ryuta Murakami [1], Yusuke Morita [2] and Atsushi Yoshimori [3]

Table S1: Enrichment factors for 52 GPCRs which were calculated from the results of screening using virtual orphan (VO) and half-sampled (HS) GPCR models. Values are shown for all combination of descriptors we used: alvaDesc and ECFP as compound descriptors, and PROFEAT2016, MSA, and ProtVec as protein descriptors. The underlined values in VO columns indicate higher values for VO models than those of the HS models.

Gene name	alvaDesc						ECFP					
	PROFEAT2016		MSA		ProtVec		PROFEAT2016		MSA		ProtVec	
	VO	HS	VO	HS	VO	HS	VO	HS	VO	HS	VO	HS
ADRA1A	9.66	32.11	13.05	29.11	8.39	27.66	2.94	19.55	10.05	28.33	5.22	11.00
ADRA1B	13.05	21.34	23.57	30.33	16.63	21.20	4.63	9.55	8.28	14.18	10.31	13.90
ADRA1D	12.93	21.34	21.62	25.29	17.60	25.14	3.65	19.15	9.57	24.27	1.17	18.42
ADRB1	4.60	75.67	44.56	82.14	10.67	72.34	4.21	67.44	32.80	80.96	3.43	68.81
ADRB2	0.78	67.83	30.21	67.44	0.06	62.64	3.70	70.42	26.84	71.59	1.23	67.05
ADRB3	5.09	81.09	19.36	62.07	10.05	62.21	3.80	62.48	22.28	56.78	15.55	92.36
AGTR1	2.48	89.10	1.20	89.87	5.74	88.50	1.11	97.93	1.03	90.21	0.86	95.36
AGTR2	3.33	70.22	2.33	52.22	2.11	44.22	2.00	33.33	4.00	10.67	3.89	12.44
CCKBR	0.39	76.91	7.59	83.41	0.00	82.63	9.86	42.20	13.41	53.64	4.93	56.79
CCR2	2.03	61.96	2.97	44.12	0.94	42.08	3.26	9.43	5.66	22.20	3.41	24.09
CCR5	1.89	72.65	1.60	56.63	0.63	47.94	2.80	58.35	1.77	38.67	0.57	44.16
CHRM1	16.06	38.46	19.45	40.27	4.13	32.24	9.33	16.63	11.31	15.04	2.09	13.23
CHRM2	29.20	38.59	21.50	32.70	10.58	30.42	12.32	24.26	12.12	22.79	5.83	15.82
CHRM3	13.98	27.00	43.69	42.60	28.57	32.52	15.42	21.84	20.82	30.24	19.74	14.16
CHRM4	13.98	42.13	25.03	47.73	20.50	31.20	5.59	10.40	17.17	17.60	8.65	29.06
CHRM5	30.39	59.61	29.01	61.96	39.60	58.82	12.74	50.98	14.31	48.23	14.70	44.31
CRHR1	0.42	72.55	1.27	80.56	0.42	65.76	0.12	72.19	4.00	80.44	1.27	76.07
CXCR3	8.37	65.39	7.28	37.34	3.37	36.07	1.18	12.93	1.55	14.75	0.73	5.65
EDNRA	3.26	73.02	32.96	78.38	2.76	63.81	2.51	44.88	15.31	60.12	3.60	44.21
FFAR1	0.13	87.33	0.39	75.96	0.00	76.73	3.23	38.75	0.39	36.17	0.13	55.29
GHSR	1.10	5.06	2.53	17.14	0.39	8.44	2.53	1.95	3.70	4.03	2.66	1.56
GLP1R	0.06	10.54	0.14	9.50	0.03	9.67	2.43	7.30	2.52	8.05	0.14	8.40
GNRHR	4.27	80.41	1.64	67.91	0.82	53.11	12.24	15.79	11.17	9.54	12.32	21.54
GPR119	0.00	20.09	0.16	20.42	0.00	8.26	2.27	5.19	2.27	7.94	1.94	2.27
GPR55	0.00	34.21	0.00	39.47	0.00	13.16	0.00	30.26	0.00	31.58	0.00	34.21
HCRTR1	3.41	55.81	37.08	58.26	6.54	44.54	0.50	31.90	20.36	48.81	4.32	35.63
HCRTR2	5.21	65.35	25.77	46.43	3.18	54.01	0.61	29.11	14.44	31.79	0.00	41.52
HRH1	10.31	46.68	7.11	35.46	7.51	27.65	3.00	26.05	5.50	22.44	3.30	25.65
HRH3	0.53	30.70	0.44	31.05	0.24	29.69	0.94	28.22	0.47	22.92	0.29	37.77
HRH4	0.33	80.71	0.22	80.26	0.00	67.18	0.11	79.16	0.33	80.93	0.00	77.60
HTR1A	9.77	25.42	12.82	26.61	3.14	21.46	4.13	27.29	8.80	26.50	7.44	28.54
HTR1B	6.11	46.35	11.05	26.82	20.08	20.81	9.87	26.61	12.01	27.04	6.87	45.92
HTR1D	9.46	44.33	9.27	18.55	4.82	20.03	11.78	28.56	14.93	26.71	11.50	48.04
HTR2A	5.73	22.37	15.17	19.89	10.93	21.41	3.98	22.71	11.49	26.16	8.61	23.56
HTR2B	2.24	22.00	10.99	20.21	5.01	16.32	0.37	8.53	3.51	3.74	2.32	10.63
HTR2C	6.22	30.06	11.36	27.51	8.77	23.03	3.90	21.87	7.96	15.69	6.22	17.62
HTR6	0.68	59.16	4.41	58.07	0.92	53.55	0.07	56.36	2.26	58.82	0.27	38.57
HTR7	11.09	36.29	19.64	27.15	6.92	27.67	0.91	27.28	10.51	29.89	4.70	31.20
MC4R	38.24	88.57	43.39	82.51	20.98	71.17	33.31	88.22	31.54	69.18	20.07	78.87
MCHR1	5.97	58.09	7.38	61.62	5.04	43.84	0.06	32.99	1.41	33.37	0.29	37.87
NPY5R	1.54	18.30	0.96	19.65	0.10	11.37	0.67	15.22	0.19	10.79	0.00	16.37
OPRD1	18.14	81.18	37.82	77.22	16.13	68.35	18.30	69.30	26.63	61.81	21.95	56.47
OPRK1	18.43	41.20	35.26	40.94	23.13	43.02	14.56	30.62	25.32	37.84	19.58	37.25
OPRL1	3.45	25.15	9.35	30.52	2.38	17.18	3.37	27.45	5.29	25.61	3.83	15.18
OPRM1	18.67	69.80	38.18	61.69	19.09	59.79	17.12	52.41	36.52	48.09	24.38	43.30
P2RY12	0.44	75.22	0.11	80.26	0.00	69.95	0.00	82.01	0.11	76.97	0.00	84.87
PTGDR2	0.55	74.32	0.51	44.80	0.00	29.76	0.04	19.53	0.00	25.43	0.00	13.23
S1PR1	8.13	57.76	16.44	68.48	5.50	44.82	10.25	30.31	13.67	35.30	3.32	39.18
TACR1	10.11	37.53	10.07	33.16	2.92	22.36	6.30	43.44	3.64	39.41	4.58	40.87
TACR2	10.33	53.14	10.70	42.06	3.78	48.35	5.16	35.01	14.35	29.97	2.64	61.20
TACR3	28.42	78.92	27.53	59.63	21.57	64.20	7.36	12.69	17.89	14.21	10.66	16.75
TSHR	0.88	4.39	0.53	4.74	1.32	5.09	0.00	3.86	0.00	5.09	0.35	6.49

Table S2: AUROC for 52 GPCRs which were calculated from the results of screening using virtual orphan (VO) and half-sampled (HS) GPCR models. Values are shown for all combination of descriptors we used: alvaDesc and ECFP as compound descriptors, and PROFEAT2016, MSA, and ProtVec as protein descriptors.

Gene name	alvaDesc						ECFP					
	PROFEAT2016		MSA		ProtVec		PROFEAT2016		MSA		ProtVec	
	VO	HS	VO	HS	VO	HS	VO	HS	VO	HS	VO	HS
ADRA1A	0.8044	0.9388	0.8778	0.9428	0.8362	0.9403	0.7665	0.9137	0.8600	0.9399	0.8329	0.8980
ADRA1B	0.8796	0.9333	0.9442	0.9528	0.9334	0.9430	0.8066	0.9275	0.9093	0.9443	0.8755	0.9172
ADRA1D	0.8730	0.9259	0.9164	0.9466	0.8825	0.9161	0.8238	0.9225	0.9045	0.9414	0.8369	0.9122
ADRB1	0.8140	0.9441	0.9135	0.9562	0.8837	0.9388	0.7887	0.9525	0.9478	0.9692	0.8134	0.9533
ADRB2	0.6870	0.8868	0.8136	0.8820	0.5998	0.8799	0.6964	0.9095	0.8621	0.8927	0.6606	0.8733
ADRB3	0.7629	0.9794	0.7659	0.9744	0.7602	0.9642	0.7776	0.9768	0.8472	0.9805	0.7999	0.9846
AGTR1	0.7573	0.9845	0.8192	0.9892	0.8327	0.9874	0.6514	0.9984	0.7200	0.9950	0.7763	0.9975
AGTR2	0.7773	0.9444	0.8515	0.9504	0.8275	0.9524	0.7433	0.9678	0.8412	0.9443	0.8552	0.9564
CCKBR	0.7313	0.9393	0.7761	0.9628	0.7157	0.9608	0.7686	0.9426	0.7940	0.9532	0.7106	0.9644
CCR2	0.8418	0.9668	0.8614	0.9638	0.8055	0.9514	0.8030	0.9088	0.8346	0.9291	0.8259	0.9527
CCR5	0.7647	0.9838	0.7289	0.9758	0.6343	0.9696	0.7261	0.9737	0.6783	0.9558	0.6446	0.9720
CHRM1	0.7844	0.8983	0.7807	0.9183	0.7328	0.9084	0.7616	0.8882	0.7687	0.8859	0.6820	0.8516
CHRM2	0.9209	0.9303	0.9167	0.9455	0.8641	0.9224	0.8999	0.9338	0.9011	0.9338	0.8278	0.9132
CHRM3	0.9116	0.9515	0.9311	0.9607	0.9141	0.9472	0.9084	0.9340	0.9294	0.9462	0.8877	0.9016
CHRM4	0.7939	0.9257	0.8071	0.9330	0.8087	0.9294	0.7567	0.8844	0.8167	0.9166	0.7733	0.8873
CHRM5	0.8856	0.9274	0.8847	0.9235	0.8896	0.9162	0.8666	0.9068	0.8673	0.8946	0.8612	0.8974
CRHR1	0.6617	0.9591	0.6588	0.9703	0.6094	0.9658	0.7760	0.9734	0.7643	0.9723	0.8237	0.9733
CXCR3	0.8333	0.9652	0.8105	0.9464	0.7874	0.9429	0.7835	0.8737	0.7463	0.8692	0.6717	0.8725
EDNRA	0.6942	0.9456	0.7374	0.9563	0.6752	0.9305	0.6848	0.9485	0.7353	0.9695	0.7000	0.9322
FFAR1	0.5006	0.9775	0.4878	0.9572	0.4546	0.9642	0.6438	0.9590	0.6308	0.9465	0.5930	0.9646
GHSR	0.7460	0.9241	0.7664	0.9160	0.6813	0.9080	0.7515	0.8009	0.7623	0.8622	0.6701	0.8179
GLP1R	0.3864	0.5672	0.4126	0.5611	0.3616	0.5523	0.3906	0.5842	0.4127	0.5907	0.3824	0.6090
GNRHR	0.8721	0.9854	0.8311	0.9738	0.8001	0.9711	0.8627	0.9275	0.8158	0.9252	0.8382	0.9570
GPR119	0.6788	0.9256	0.6348	0.9302	0.6506	0.9032	0.7148	0.8791	0.6782	0.8691	0.6747	0.8583
GPR55	0.4432	0.7032	0.4211	0.6772	0.3385	0.6148	0.4476	0.7076	0.4684	0.6756	0.3418	0.7217
HCRTR1	0.8063	0.9378	0.8643	0.9442	0.8281	0.9328	0.8145	0.9279	0.8605	0.9319	0.8125	0.9305
HCRTR2	0.7472	0.9700	0.8363	0.9596	0.7437	0.9640	0.7658	0.9459	0.8435	0.9521	0.7414	0.9608
HRH1	0.7585	0.9263	0.7651	0.9315	0.7945	0.9239	0.7598	0.9287	0.7728	0.9017	0.7697	0.9084
HRH3	0.5726	0.9509	0.4812	0.9507	0.4409	0.9454	0.6307	0.9566	0.5672	0.9521	0.6017	0.9652
HRH4	0.4868	0.9576	0.5128	0.9561	0.4062	0.9444	0.5193	0.9647	0.5887	0.9730	0.4010	0.9544
HTR1A	0.8850	0.9480	0.8799	0.9490	0.8003	0.9387	0.8525	0.9555	0.8541	0.9536	0.8673	0.9590
HTR1B	0.8435	0.9626	0.9146	0.9635	0.8184	0.9458	0.8853	0.9532	0.9218	0.9621	0.8867	0.9639
HTR1D	0.8773	0.9457	0.9081	0.9518	0.8129	0.9404	0.9017	0.9392	0.9205	0.9551	0.8932	0.9591
HTR2A	0.8210	0.9263	0.8790	0.9302	0.8593	0.9321	0.8047	0.9263	0.9001	0.9446	0.8624	0.9369
HTR2B	0.7131	0.9042	0.8159	0.9053	0.7716	0.9008	0.6515	0.8493	0.7912	0.8673	0.7386	0.8705
HTR2C	0.7951	0.9108	0.8585	0.9074	0.8211	0.9035	0.7642	0.9056	0.8677	0.8991	0.8139	0.8997
HTR6	0.5503	0.9668	0.6181	0.9683	0.4978	0.9616	0.4719	0.9660	0.5324	0.9730	0.4460	0.9477
HTR7	0.8382	0.9495	0.8972	0.9432	0.8162	0.9473	0.7912	0.9469	0.8686	0.9565	0.8207	0.9544
MC4R	0.9185	0.9870	0.9108	0.9848	0.8557	0.9795	0.9386	0.9940	0.9338	0.9905	0.8996	0.9924
MCHR1	0.7478	0.9760	0.7451	0.9776	0.7322	0.9677	0.8069	0.9623	0.7778	0.9594	0.7842	0.9639
NPY5R	0.5750	0.9221	0.5218	0.9209	0.5522	0.9216	0.6105	0.8953	0.6663	0.8846	0.5764	0.8828
OPRD1	0.8954	0.9725	0.9219	0.9779	0.8751	0.9681	0.9103	0.9766	0.9402	0.9796	0.9068	0.9731
OPRK1	0.8498	0.9523	0.8516	0.9557	0.8197	0.9450	0.8461	0.9387	0.8746	0.9587	0.8400	0.9560
OPRL1	0.7467	0.9485	0.8231	0.9594	0.6872	0.9296	0.7933	0.9537	0.8827	0.9351	0.7830	0.9334
OPRM1	0.9131	0.9701	0.9220	0.9640	0.9025	0.9636	0.9138	0.9737	0.9370	0.9730	0.9268	0.9686
P2RY12	0.7515	0.9542	0.7191	0.9653	0.6737	0.9394	0.6491	0.9728	0.5593	0.9740	0.7506	0.9683
PTGDR2	0.5548	0.9780	0.6064	0.9530	0.4303	0.9347	0.5927	0.9059	0.6316	0.9171	0.4541	0.9258
S1PR1	0.7021	0.9693	0.6409	0.9764	0.5902	0.9496	0.7649	0.9461	0.6798	0.9567	0.6956	0.9629
TACR1	0.8151	0.9581	0.7761	0.9553	0.7746	0.9358	0.7858	0.9727	0.7337	0.9589	0.7384	0.9670
TACR2	0.9000	0.9716	0.8787	0.9660	0.8491	0.9515	0.8643	0.9635	0.8526	0.9544	0.8503	0.9616
TACR3	0.6954	0.9679	0.6650	0.9512	0.6619	0.9430	0.7313	0.9360	0.7000	0.9055	0.6759	0.9326
TSHR	0.4271	0.5035	0.4403	0.5148	0.4817	0.5236	0.3619	0.5226	0.3935	0.5086	0.4283	0.5629
