# Supplementary figures and images for: Computational Prediction of Compound–Protein Interactions for Orphan Targets Using CGBVS
Source: Molecules. 2021 Aug 24;26(17):5131. doi: 10.3390/molecules26175131 (PMC8434178; doi:10.3390/molecules26175131)

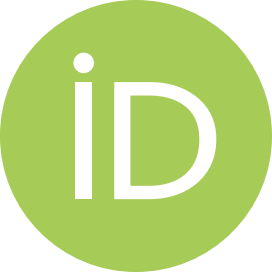

Supplement: Supplementary file 1 [file molecules-26-05131-s001.zip › Definitions/logo-orcid-eps-converted-to.pdf]

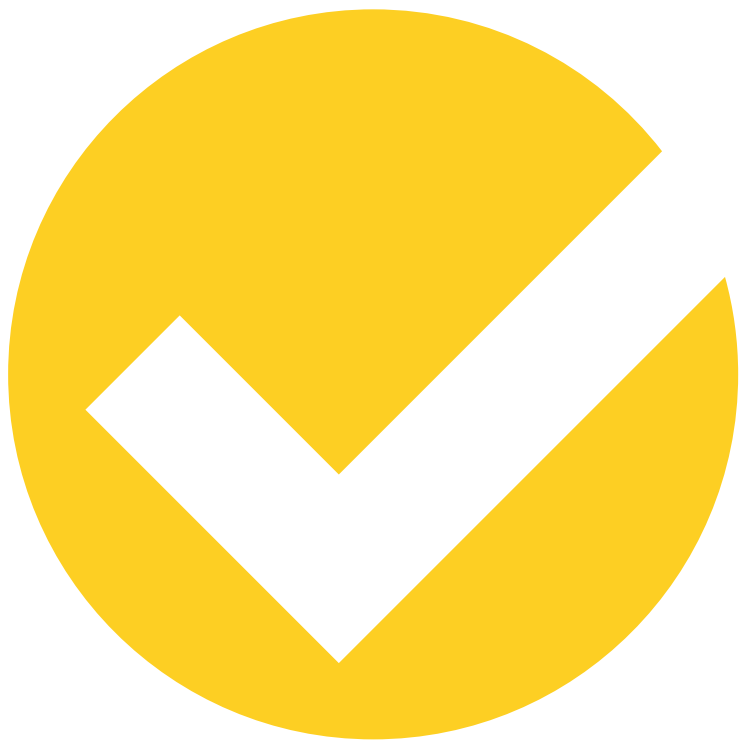

check for  
updates

Supplement: Supplementary file 1 [file molecules-26-05131-s001.zip › Definitions/logo-updates.pdf]
